# 微转移在非小细胞肺癌的预后价值

**DOI:** 10.3779/j.issn.1009-3419.2013.09.10

**Published:** 2013-09-20

**Affiliations:** 1 510080 广州，广东省肺癌研究所，广东省人民医院，广东省医学科学院 Guangdong Lung Cancer Institute, Guangdong Genral Hospital & Guangdong Academy of Medical Sciences, Guangzhou 510080, China; 2 510515 广州，南方医科大学 Southern Medical University, Guangzhou 510515, China

**Keywords:** 肺肿瘤, 微转移, 预后, Lung neoplasms, Micrometastasis, Prognosis

## Abstract

肺癌是我国目前发病率和死亡率最高的恶性肿瘤。非小细胞肺癌（non-small cell lung cancer, NSCLC）占肺癌的80%-85%。诊断时的分期是NSCLC主要的预后因子，也是治疗模式选择的重要依据。然而，完全性切除术后的Ⅰ期的NSCLC仍有25%-30%的复发率。这部分复发的患者可能早期就存在局部和（或）远处的隐匿性转移。因此，很多研究开始对NSCLC患者进行微转移的检测，并评估其预后价值。本文总结了近年来的相关研究，并就NSCLC的淋巴结微转移、骨髓微转移、胸膜腔微转移及外周血微转移的预后作用进行综述。

## Abstract (en)

Lung cancer ranks top in morbidity and mortality among the malignancies in China. Non-small cell lung cancer (NSCLC) accounts for about 80%-85% of all lung malignancies. The stage at diagnosis is the principal prognostic indicator, and accurate staging is essential for treatment decisions. After radical surgical resection in patients with stage Ⅰ disease, the recurrence rate ranges from 25% to 30%. Patients with a poor outcome may have occult locoregional and/or distant metastasis before resection. In recent years, there are a lot of researches aimingat detecting micrometastasis and evaluating the prognostic value of it. This study reviewed the prognostic value of micrometastasis in lymph node, bone marrow, pleural cavity and peripheral blood.

肺癌是我国目前发病率和死亡率最高的恶性肿瘤^[1]^。非小细胞肺癌（non-small cell lung cancer, NSCLC）占肺癌的80%-85%，分子靶点的发现及相应靶向药物的临床应用进一步开辟了NSCLC个体化治疗的时代，但总体上NSCLC的5年生存率仍滞留在15%-20%左右。完全性切除的Ⅰ期NSCLC，常规临床方法检测不到残余肿瘤细胞的患者中仍有25%-30%患者最终会发生局部复发或远处转移^[2, 3]^，这可能与肺癌患者早期即存在全身微转移有关。这些微转移灶存在于外周血、骨髓、淋巴结或浆膜腔中，在一定条件下增殖浸润，引起肿瘤复发、转移。NSCLC的微转移主要包括淋巴结微转移、骨髓腔微转移、胸膜腔微转移及外周血微转移。分子生物学技术的发展将微转移的检测能力提高到了细胞和分子水平，也使检测NSCLC微转移灶用于预测疗效和判定预后成为可能。

## 1 微转移的概念

1869年，Ashworth首先报道了在1例肿瘤患者体内检测到循环肿瘤细胞（circulating tumor cells, CTCs）。肿瘤的微转移（mircrometastasis）或隐匿性转移（occult metastasis）是指恶性肿瘤在发展过程中，癌细胞播散并存活在淋巴结、骨髓、外周血及各组织器官中，但尚未形成显性转移灶，转移部位也无任何临床表现，采用影像学和常规病理方法难以检测到的微量转移，需要通过分子生物学的技术予以诊断^[4]^。

## 2 NSCLC微转移的检测方法

### 2.1 免疫组织化学法（immunohistochemistry, IHC）

IHC是利用抗原与抗体间的特异性结合原理和特殊的标记技术，对组织和细胞内的特定抗原或抗体进行定位、定性或定量检测的一门技术。IHC主要用于NSCLC淋巴结及骨髓中微转移灶的检测。IHC常用的单克隆抗体主要是抗上皮来源的蛋白，如抗上皮细胞粘附分子（EpCAM）抗体Ber-Ep4、抗细胞角蛋白家族（CK）抗体AE1/AE3。IHC诊断NSCLC微转移具有较高的敏感性，可达10^-5^-10^-6^，而且检测方法简便；缺点是由于酶染色和非特异性抗原的表达而导致特异性较低。

### 2.2 聚合酶链式反应（polymerase chain reaction, PCR）

PCR是体外酶促合成、扩增特异基因片段的一种方法，它能使目标DNA片段呈指数扩增。目前在淋巴结微转移检测中应用较多的是逆转录PCR，它是以肿瘤组织特异性表达的mRNA为分子标记，先合成cDNA后再进行PCR扩增，通过检测肿瘤标志物mRNA来反应肿瘤细胞的存在。荧光实时定量PCR（real-time fluorescent quantitative PCR）是在PCR反应体系中加入荧光基因，利用荧光信号累积实时监测整个PCR过程，通过加入已知浓度的标准样品绘制的标准曲线，能推算出样品中初始模板的浓度，可定性、定量的检测微量肿瘤细胞。荧光实时定量PCR是目前最敏感的检测方法^[5]^，敏感性可达10^-7^，与逆转录PCR相比，它可以用正常组织作对照来设定合理的cut-off值，在一定程度上减少了假阳性率。PCR主要用于检测NSCLC淋巴结、骨髓及外周血中的微转移灶，常用的分子标志物有细胞角蛋白家族（CKs）、黏蛋白-1（mucin1, MUC1）、LUNX（lung-specific x gene）、CEA、p53、Kras、端粒酶逆转酶（human telomerase reverse transcriptase, hTERT）和肺泡表面蛋白。

### 2.3 流式细胞术（ﬂow cytometry, FCM）

流式细胞术也称荧光激活细胞分离术，采用荧光标记相应抗体来检测骨髓和外周血中的肿瘤细胞。其优点是操作简单、快速、数据精确。缺点是FCM不能提供细胞形态方面的信息，无法区分带有同样标志物的肿瘤细胞和正常细胞。FCM常用于骨髓及外周血中肿瘤细胞的检测。FCM检测靶细胞的敏感度仅为10^-5^，在使用时很大程度上依赖于可分析的细胞数目，因此极大地限制了其广泛应用。

### 2.4 CellSearch系统

CellSearch系统集免疫磁珠富集技术和免疫荧光技术于一体，先将细胞富集后再进行自动化检测，其生物学原理是依据肿瘤细胞与正常细胞表达的表面蛋白不同将二者区分开来。来源于中胚层的白细胞表达CD45，来源于外胚层的癌细胞表达上皮细胞粘附分子（epithelial cell adhesion molecule, EpCAM）和细胞角蛋白（cytokeratin, CK），但不表达CD45。该系统首先利用免疫磁珠技术将EpCAM标记的CTCs从血液样本中富集出来，然后加入荧光抗体标记和化学染色来准确识别CTCs，系统将“EpCAM^+^CK^+^DAPI^+^CD45^-^”的细胞界定为CTCs。CellSearch系统可以检测7.5 mL血液样品（约含400多亿的血细胞）中单一的CTCs，是目前自动化程度最高的CTCs检测技术，且受人为因素影响较小，具有较高的特异性、敏感性及可重复性^[6]^，该系统是目前唯一被美国食品药品监督管理局（FDA）批准用于转移性乳腺癌、结直肠癌和前列腺癌CTCs检测的新技术^[7-9]^。近年来，应用该系统检测肺癌及其他实体瘤CTCs的研究也越来越受重视。但该检测系统会遗漏缺失上皮细胞表面标志的肿瘤细胞，例如经过上皮间质转化（epithelial-mesenchymal transition, EMT）后的细胞，而且检测费用较高，限制了其在临床上广泛应用。

### 2.5 其他方法

蛋白免疫印迹（Western blot or Immunoblotting），是根据抗原抗体的特异性结合检测复杂样品中的某种蛋白的方法，现已成为蛋白分析的一种常规技术，具有分辨率高、特异性强和敏感度高等优点。目前该方法在微转移检测上应用较少，Chen等^[10]^曾报道使用Western blot检测NSCLC淋巴结微转移。此外，也有研究应用基因表达谱技术^[11]^和蛋白质组学技术^[12]^检测NSCLC淋巴结微转移。

## 3 微转移与NSCLC预后

### 3.1 淋巴结微转移与NSCLC预后

淋巴结是否受累是包括NSCLC在内的多数实体瘤术后无瘤生存期（disease free survival, DFS）和总生存期（overall survival, OS）的重要预测因子。应用IHC或逆转录PCR对常规病理方法检查阴性的淋巴结进行微转移检测，有助于对患者进行更准确的“分子分期”，在预测复发和评估预后上均有着重要意义，同时能够为制定多学科综合治疗方案提供更准确的依据。周清华等^[13]^的研究通过对比常规病理组织学方法和逆转录PCR诊断肺癌微转移，证实逆转录PCR敏感性更强，灵敏度更高，能提高淋巴结和循环系统微转移的检出率。杨浩贤等^[14]^用逆转录PCR方法检测淋巴结中LUNX mRNA的表达，也证实NSCLC患者的纵隔淋巴结中，有常规病理学无法诊断的微转移灶存在。

Kubuschok等^[15]^报道，用IHC检测127例患者的淋巴结微转移，检出25例患者存在淋巴结微转移，随访64个月，发现检测出微转移的患者其肿瘤复发率较无微转移者高出2.7倍，OS风险比HR=2.5。Le Pimpec-Barthes等^[16]^用逆转录PCR的检测淋巴结中CK19 mRNA。在未发生微转移的患者，术后2年生存率为100%，而伴有微转移的患者，2年生存率仅为64.5%，差异有统计学意义（*P*=0.04）。欧阳伟炜等^[17]^应用IHC检测78例Ⅰ期NSCLC根治术后区域淋巴结的微转移，并评估淋巴结微转移与患者长期生存的关系。淋巴结微转移检出率为26.9%（21/78），伴有微转移的患者中位生存期为23个月，不伴有微转移的患者为87个月，差异有统计学意义（*P*=0.008）。

迄今，检测淋巴结微转移最大样本量的前瞻性临床试验ACOSOG Z0040^[18]^的研究结果再次证实了淋巴结微转移的预后价值。经HE染色确认为N0的NSCLC患者中，淋巴结微转移组与未发生微转移组相比，术后DFS（HR=1.63, *P*=0.009）和OS（HR=1.59, *P*=0.007）均有统计学差异。然而发生微转移的淋巴结数目及淋巴结站与患者OS无相关性。多变量分析显示，淋巴结微转移是预后的独立危险因素。

综合分析（表 1），虽然有少数研究^[19-21]^未证实淋巴结微转移与不良预后有明确关系，但大量研究表明淋巴结微转移是NSCLC患者预后的独立危险因素。在今后的临床实践中，我们可以考虑通过检测淋巴结微转移，对患者进行危险因素分层，复发风险高的患者可进行术后的辅助治疗^[22]^。

**1:** 探讨淋巴结微转移预后作用的主要临床研究 Major clinical studies of the prognostic value of lymph node micrometastasis

Study (Year)	Number of patients	Method/Marker	Frequency of positivity in		Prognostic relevance
			Histology negative nodes	Patients with pN0 disease	
Chen *et al* (1993)^[38]^	65	IHC/Polyclonal (antikeratin)	102/588 (17.0%)	38/60 (63.0%)	Trend toward shorter survival time
Kubuschok *et al* (1999)^[15]^	125	IHC/Ber-Ep4	35/565 (6.2%)	11/70 (16.0%)	Worse OS (*P* < 0.000, 1); Worse DFS (*P* < 0.000, 1)
Hashimoto *et al* (2000)^[39]^	31	MASA/P53, K-ras mutation	47/170 (28.0%)	6/22 (27.0%)	Worse disease-specific survival
Osaki *et al* (2002)^[27]^	115	IHC/AE1, AE3	42/2, 432 (1.7%)	32/115 (28.0%)	Worse OS; Higher RR (*P*=0.01)
Tezel *et al* (2006)^[40]^	21	IHC/Ber-Ep4, AE1, E3	NA	NA	Reduced DFS (*P*=0.002)
Rena *et al* (2007)^[19]^	87	IHC/AE1, AE3	19/694 (2.7%)	14/87 (16.0%)	No significance
Li *et al* (2008)^[41]^	89	RT-PCR/MUC1 mRNA	36/402 (9.0%)	21/89 (23.6%)	Worse OS (*P* < 0.05)
Ouyang *et al* (2008)	78	IHC/AE1, AE3	NA	21/78 (26.9%)	Worse OS (*P*=0.008)
Yamashita *et al* (2010)^[42]^	117	IHC/cytokeratin	NA	34/117 (29.1%)	Poor survival (*P* < 0.001)
Rusch *et al* (2011)^[18]^	1047	IHC/cytokeratin	NA	130/580 (22.4%)	Worse DFS (*P*=0.009); Worse OS (*P* < 0.007)
Li *et al* (2013)^[43]^	44	RT-PCR/surviving and livin mRNA	79/286 (27.6%)	15/44 (34.1%)	Worse TFS (*P*=0.007); Worse OS (*P*=0.01)
Dai *et al* (2013)^[44]^	49	RT-PCR/FHIT and CDKN2A transcript deletion	FHIT: 39/176 (22.0%); CDKN2A: 22/116 (19.0%)	16/49 (32.7%)	Reduced DFS (*P*=0.001); Reduced OS (*P*=0.002)
MASA: mutant allele specific amplification; NA: not available; TFS: tumor-free survival; OS: overall survival; DFS: disease free survival.					

### 3.2 骨髓微转移与NSCLC预后

骨髓组织内存在大量网状结缔组织，可以对血流中的癌细胞起过滤作用，释放入血的癌细胞经过骨髓即被滤下，理论上骨髓中的弥散肿瘤细胞（disseminated tumour cells，DTCs）是比较理想的微转移检测指标。

目前，骨髓微转移检测的取材部位主要是肋骨和髂骨，有研究比较了两个部位微转移的检出率，证实肋骨的检出率更高^[23, 24]^。骨髓微转移的发生率与NSCLC的TNM分期无关^[24-26]^。

关于骨髓微转移的预后价值，各研究结果互为矛盾（表 2），究其原因在于研究方法的不统一和研究例数不足，且大部分研究未设计正常对照组。ACOSOG Z0040^[18]^研究显示，骨髓微转移发生与淋巴结微转移无关，患者的预后与骨髓微转移发生无相关性。至此，我们不禁产生一个疑问：骨髓腔中的DTCs是真的转移灶，还是仅仅是我们看到的一个表象呢？很多假设试图解释这一现象，Osaki等^[27]^检测了同一批患者的淋巴结和骨髓微转移，发现这两个部位微转移之间没有相关性，这可能与不同部位微转移的发生的机制不同有关；也有观点认为，骨髓腔的DTCs大部分处于休眠期，这些休眠细胞要活化并形成显性转移灶，需要转移部位微环境、DTCs自身突变和患者遗传特性三者的综合作用^[28]^。目前，骨髓微转移的预后价值尚无定论。

**2:** 探讨骨髓微转移预后作用的主要临床研究 Major clinical studies of the prognostic value of DTC in bone marrow

Study (Year)	Number of patients	Site of bone marrow harvest	Marker	Median follow up (month)	Frequency of occult disease	Prognostic relevance
Pantel *et al* (1996)^[45]^	139	Iliac crest and rib	CK2	39.0	83/139 (59.7%)	Earlier recurrence (*P*=0.028)
Hsu *et al* (2000)^[25]^	96	Iliac crest	AE1/AE3, MNF116, Ber-Ep4	20.2	21/96 (22.0%)	NS in 30 months OS or DFS (*P*=0.94; *P*=0.57)
Osaki *et al* (2002)^[46]^	115	Iliac crest	CK2	35.8	32/115 (28.0%)	NS in OS
Yasumoto *et al* (2003)^[47]^	351	Iliac crest	CK2	NA	112/351 (31.9%)	Worse OS with stage Ⅱ to Ⅲa (*P*=0.076); NS with stage Ⅰ
Nosotti *et al* (2008)^[48]^	87	Rib	Cytokeratin	35.3	14/87 (16.0%)	Predictor of cancer recurrence (HR=2.09, *P*=0.002, 6); NS on DFS
Brunsvig *et al* (2008)^[26]^	196	Iliac crest	MOC31 (anti-EpCam)	8.0	107/196 (55.0%)	NS
Ruffato *et al* (2009)^[49]^	68	Rib	MNF116	61.0	17/68 (25.0%)	Worse free from recurrence survival rate in stage Ⅰa and Ⅰ b
Rusch *et al* (2011)^[18]^	1047	Rib	Cytokeratin	60.0	66/821 (8.0%)	NS
NS: no siginificance; NA: not available.						

### 3.3 胸膜腔微转移与NSCLC预后

胸膜腔微转移可能是造成NSCLC患者术后并发恶性胸腔积液及肿瘤复发的直接原因。通过胸腔灌洗液细胞学（pleural lavage cytology, PLC）检查来评价胸膜腔微转移的研究已经进行了半个世纪^[29-31]^，但大多数研究样本量小，预后价值尚不明确。

为了明确胸膜腔微转移的预后价值，国际胸腔灌洗液细胞学研究者联盟初筛了目前已经发表研究结果31篇相关论文，最终共有8, 736例患者被纳入*meta*分析，511（5.8%）例患者PLC阳性。与PLC阴性患者相比，PLC阳性者预后更差，风险比HR=1.465（95%CI: 1.290-1.665; *P* < 0.001）。PLC阳性是预后的独立危险因素^[32]^。Srdjan Saso等^[33]^认为术前PLC阳性是患者术后发生胸膜、远处及全身复发的预测因子，同时PLC阳性预示着NSCLC患者尤其是Ⅰ期NSCLC患者预后更差。基于以上假设，他们筛选了2011年以前收录在Medline、EMBASE和Google Scholar三个数据库的相关研究，最终共4, 450例患者被纳入分析。结果显示，术前PLC阳性患者与阴性患者相比，胸膜、远处及全身复发的风险更高（OR=4.82, 95%CI: 2.45-9.51），随访显示，PLC阳性组与阴性组相比，OS风险比HR=2.08（95%CI: 1.71-2.52, *P* < 0.000, 01）。单独分析9项研究中的Ⅰ期患者，OS风险比HR=4.2（95%CI: 2.65-6.65, *P* < 0.02）。

然而，ACOSOG Z0040^[18]^的研究结果却未能证实以上结论，术前和术后PLC阳性率分别为3.3%（29/885）和2.0%（17/871），PLC性质与患者OS无相关性。

如何解释这一争议？研究结果的矛盾性一方面与纳入*meta*分析的各个研究并没有采用标准的灌洗方法且大多数研究的随访时间都少于3年有关；另一方面可能与ACOSOG Z0040 PLC阳性检出率过低，使得阳性组和阴性组的可比性差有关。

### 3.4 外周血微转移与NSCLC预后

肿瘤细胞脱落、侵袭并进入血液循环是实现肿瘤转移的最初阶段。杨浩贤等^[34]^用逆转录PCR检测肺癌患者和肺良性疾病患者外周血中的LUNX mRNA，肺良性病变患者外周血中未见LUNX-mRNA表达，4例术前和术中LUNX mRNA表达阳性的患者，术后1周转为阴性，提示外周血中LUNX mRNA来源于肺的原发肿瘤细胞。周清华等^[35]^用逆转录PCR，检测患者外周血中CK19 mRNA表达，提示外周血“微转移”是预测局部晚期NSCLC预后的独立因素。

近年来，许多研究通过计算外周血中CTCs数目来检测外周血微转移。在肺癌患者中，CTCs数量与肿瘤进展呈正相关，尤其伴随肿瘤远处转移灶的增多而升高。检测肿瘤患者外周血CTCs可早期评估肿瘤转移风险，预测肿瘤复发和判断患者预后。

Hofman等^[36]^检测了208例可手术NSCLC患者和39例健康对照者的外周血。他们采用ISET法检测外周血中非血细胞的循环细胞（circulating nonhematologic cells, CNHCs)。受试者平均随访24个月。上述患者中，CNHCs平均个数是40。CNHCs≥50和 < 50的患者相比，OS和DFS均更短，差异有统计学意义（*P*=0.002和*P*=0.001）。单因素和多因素分析均显示，排除疾病分期的影响，术前CNHCs数目≥50预示着更短的DFS和OS。Krebs等^[37]^通过CellSearch系统检测101例局部晚期和晚期NSCLC患者外周血CTCs。Ⅳ期患者外周血中CTCs数目（0-146）明显高于Ⅲb期（0-3）和Ⅲa期（未检测到）。单因素分析显示，化疗前每7.5 mL外周血中CTCs < 5个和≥5个的患者的PFS分别是6.8个月和2.4个月（*P* < 0.001），OS分别是8.1个月和4.3个月（*P* < 0.001），差异有统计学意义。多因素分析显示，CTCs数量是OS的最强预测因子。

## 4 微转移检测的临床意义及研究展望

对NSCLC患者的微转移灶进行检测，一方面支持NSCLC早期的全身隐匿性播散，提示NSCLC是一种全身性疾病而非局灶性病变；另一方面，可以补充常规病理学确认的TNM分期，为更准确的“分子分期”提供依据，从而指导治疗方案的选择。目前NSCLC微转移的研究中，还存在几个问题：①尚缺乏特异性强、灵敏度高的理想检测标志物；②对特定的检测部位最优的检测方法不统一，假阳性和假阴性等问题影响了研究结论；③骨髓腔中DTCs和外周血CTCs的最佳检测时间尚不明确；④检测出有微转移的患者应采取何种治疗措施没有循证医学证据。同时，微转移对NSCLC的预后价值还存在争议，微转移的检出并不代表一定会形成显性转移灶，肿瘤细胞自身生物学特性、机体免疫状况和局部微循环等都是重要的影响因素。因此，筛选敏感性和特异性均较高的分子标志物是今后研究的一个方向。同时为得到更为可靠的研究结论, 减少假阳性结果，获取恰当的组织标本、选择合适的检测方法、建立详尽的检测过程及制定规范的质控标准极为重要。未来我们期待多中心合作，开展前瞻性的临床研究，采用统一规范的标准对肺癌微转移灶进行检测，明确微转移对NSCLC的预后价值。同时，对伴有微转移的NSCLC患者予以恰当的辅助治疗，试图消除微小残留病灶，获得生存获益。
